# Potato late blight field resistance from QTL *dPI09c* is conferred by the NB-LRR gene *R8*

**DOI:** 10.1093/jxb/ery021

**Published:** 2018-01-27

**Authors:** Rui Jiang, Jingcai Li, Zhendong Tian, Juan Du, Miles Armstrong, Katie Baker, Joanne Tze-Yin Lim, Jack H Vossen, Huan He, Leticia Portal, Jun Zhou, Merideth Bonierbale, Ingo Hein, Hannele Lindqvist-Kreuze, Conghua Xie

**Affiliations:** 1 Key Laboratory of Potato Biology and Biotechnology, Ministry of Agriculture, P. R. China, Wuhan, China; 2 National Center for Vegetable Improvement (Central China), Wuhan, China; 3 Huazhong Agricultural University, Wuhan, Hubei, China; 4 School of Life Sciences, Huanggang Normal College, Huanggang, Hubei, China; 5 Key Laboratory of Horticultural Plant Biology (HZAU), Ministry of Education, Wuhan, China; 6 Potato Engineering and Technology Research Center of Hubei Province, Wuhan, China; 7 Cell and Molecular Sciences, The James Hutton Institute, Dundee, Scotland, UK; 8 The University of Dundee, Division of Plant Sciences at the James Hutton Institute, Dundee, UK; 9 Wageningen UR Plant Breeding, Wageningen University and Research, AJ Wageningen, The Netherlands; 10 International Potato Center, Apartado, Lima, Peru

**Keywords:** dRenSeq, field resistance, late blight, map-based cloning, potato, *R* gene

## Abstract

Following the often short-lived protection that major nucleotide binding, leucine-rich-repeat (NB-LRR) resistance genes offer against the potato pathogen *Phytophthora infestans*, field resistance was thought to provide a more durable alternative to prevent late blight disease. We previously identified the QTL *dPI09c* on potato chromosome 9 as a more durable field resistance source against late blight. Here, the resistance QTL was fine-mapped to a 186 kb region. The interval corresponds to a larger, 389 kb, genomic region in the potato reference genome of *Solanum tuberosum* Group Phureja doubled monoploid clone DM1-3 (DM) and from which functional NB-LRRs *R8*, *R9a, Rpi-moc1*, and *Rpi_vnt1* have arisen independently in wild species. dRenSeq analysis of parental clones alongside resistant and susceptible bulks of the segregating population B3C1HP showed full sequence representation of *R8*. This was independently validated using long-range PCR and screening of a bespoke bacterial artificial chromosome library. The latter enabled a comparative analysis of the sequence variation in this locus in diverse Solanaceae. We reveal for the first time that broad spectrum and durable field resistance against *P. infestans* is conferred by the NB-LRR gene *R8*, which is thought to provide narrow spectrum race-specific resistance.

## Teaser

Using genetics, genomics, and diagnostic RenSeq, we demonstrate that the major NB-LRR gene *R8* explains the field resistance against potato late blight in the QTL *dPI09c*

## Introduction

Potato (*Solanum tuberosum* L.) is the third most important food crop in the world after rice and wheat in terms of human consumption. More than a billion people worldwide consume potatoes, and global crop production exceeded 382 million metric tons in 2014 (FAO, 2017). *Phytophthora infestans*, the causal agent of late blight disease, is the most devastating pathogen of potato, causing losses of approximately $6.7 billion annually (Haas *et al.*, 2009). Preventative application of chemicals is currently being used to control this disease. However, excessive use of fungicides poses detrimental risks to human health and to the environment. Moreover, isolates can become insensitive to some of the commonly used agents (Goodwin *et al.*, 1996). Thus, the characterization, cloning and introgression into cultivars of natural resistance provides an environmentally benign alternative to chemical crop protection agents.

The wild species *Solanum demissum* has been used as a donor for single race-specific resistance (*R*) genes, which mediate complete resistance to *P. infestans* isolates carrying cognate avirulence protein (Lenman *et al.*, 2016). Nonetheless, a dynamic, repeat-rich genome enables the pathogen to often evolve rapidly (Haas *et al.*, 2009; Raffaele *et al.*, 2010) and to overcome host resistance through the emergence of new pathogen races (Fry and Goodwin, 1997). Pyramiding multiple major *R* genes is considered a sustainable strategy to maintain the resistance for longer (Zhu *et al.*, 2012; Haesaert *et al.*, 2015), while deploying quantitative disease resistance (QDR) in breeding programs has also been adopted (St Clair, 2010). QDR has been described as horizontal, incomplete, field, durable, and broad-spectrum resistance by different authors owing to their interests and assumptions (Solomon-Blackburn *et al.*, 2007; Poland *et al.*, 2009). However, phenotypically, all show reduced but not absent disease symptoms. It is thought that these resistances could be more durable as the evolutionary pressure to adapt is significantly decreased for the pathogens. Therefore, QDR has been favored by potato breeders after initial simple stacking of *R* genes in the 1960s, which could be easily overcome as shown for *R1*, *R2*, and *R3a* in the cultivar Pentland Dell (Hein *et al.*, 2009).

Since the 1980s, the International Potato Center (CIP) has developed durable late blight resistant potato germplasms by excluding the known major *R* genes from selected resistant sources. The strategy was meant to eliminate the interference of *R* genes, because they could potentially mask the underlying quantitative resistance in the process of selection (Landeo, 1989). Three selection steps were carried out with complex *P. infestans* races that were predicted to overcome the major *R* genes as well as race 0, and the resulting population is known as B3 (Landeo, 1989; Landeo *et al.*, 1995). To date, the B3 population has undergone three cycles of recurrent selection to improve agronomic traits whilst maintaining broad-spectrum late blight resistance (M. Gastelo, CIP, personal communication), and a number of cultivars have been released by CIP national partners (Ndacyayisenga, 2011). To understand the genetic and molecular mechanism of the quantitative late blight resistance in the B3 population, a dihaploid population, B3C1HP, which originated from the resistant B3 advanced clone 393046.7, was used to construct a genetic map. Five independent field assessments were conducted at two locations in Peru and a major QTL, *dPI09c*, was detected on chromosome 9. The *dPI09c* QTL explained between 14.5 and 83.3% of the disease variance in different environments (Li *et al.*, 2012).

Map-based cloning has been widely used for cloning potato late blight resistance genes, such as *R1* (Ballvora *et al.*, 2002), *R2* (Lokossou *et al.*, 2009), and *RB* (Song *et al.*, 2003). Recently, with the development of high-throughput sequencing platforms, the potato genome has been sequenced (Potato Genome Sequencing Consortium, 2011; Aversano *et al.*, 2015), and this may greatly accelerate the identification of genes of interest. A total of 755 nucleotide binding, leucine-rich-repeat (NB-LRR) genes have been identified and used as probes to establish a resistance gene enrichment sequencing (RenSeq) platform (Jupe *et al.*, 2012; Jupe *et al.*, 2013) to promote the identification of *R* genes through bulked-segregation analysis. Combined with single-molecule real-time (SMRT) sequencing (SMRT RenSeq) (Witek *et al.*, 2016) or chemical mutagenesis (MutRenSeq) (Steuernagel *et al.*, 2016), RenSeq has been utilized in cloning *R* genes in different species. RenSeq has also been applied as a diagnostic tool, dRenSeq, to identify known *R* genes or their homologs (Van Weymers *et al.*, 2016). In this study, we report the molecular characterization of the *dPI09c* locus through map-based cloning, dRenSeq, allele mining, and comparative genomics.

## Materials and methods

### Plant materials

For the fine mapping of the resistance QTL, an extended progeny of the B3C1HP population was generated using the same progenitors, 301071.3 as the resistant maternal parent and 703308 as the susceptible male plant, as described by Li *et al.* (2012). Over 4000 potato true seeds were germinated *in vitro*. The seeds were sterilized with 1.5% (v/v) sodium hypochlorite solution for 15 min and rinsed three times with sterile distilled water. Seed germination was induced by incubating sterile seeds with 1000 ppm gibberellic acid for 18 h in the dark followed by culturing of seeds in plastic tubes (Falcon, 15 ml, conical) containing 5 ml of MS medium (Murashige & Skoog, 1962). The plants were multiplied in tissue culture using standard growth media for potato. Three-week-old *in vitro* plantlets were transplanted in pots containing Promix.

*Nicotiana benthamiana* was grown in the greenhouse under a 16 h/8 h light–dark cycle at 24 °C. Leaves from 5-week-old plants were used for experiments.

### DNA extraction and recombinants screening

The genomic DNA of all the 4000 progenies was extracted from 3-week-old plantlets with a fast and simple method described by Hosaka (2004). For the screening of recombinants, four markers flanking the QTL *dPI09c* region were used, namely Rpi-svnt1_367, DMC42152bf, DMC42144af, and At3g24160f2 (Li *et al.*, 2012; see Supplementary Table S1 at *JXB* online). The PCR and polyacrylamide gel running procedures were as described by Li *et al.* (2012).

### Late blight resistance evaluation

Whole plant inoculations of the 106 recombinants was conducted in a greenhouse by spraying *P. infestans* sporangia onto all recombinant plants and using Amarilis and Cruza-148 as resistant controls as well as Desiree, Yungay, and Tomasa as susceptible controls. There were six plants of each genotype and three plants of each control distributed following a randomized complete block design. Plants were 45 d old since emergence and *P. infestans* isolate PSR24 (Race 1, 2, 3, 4, 5, 6, 7, 9, 10, and 11 tested on a Black differential set) was used for the infections. This particular isolate had been collected from susceptible plants of the B3C1HP_100_ progenies in the field and purified, at a concentration of 750 sporangia ml^−1^. The inoculation was done with a hand-held sprayer until run-off. After the inoculation, plastic tents were constructed to cover the plants and maintain almost 100% humidity. Humidity was maintained high in the greenhouse by an automatic sprinkler system that switched on every 15 min. The temperature ranged between 17 and 24 °C. The disease level was evaluated by estimating the percentage area of infection in each plant 7 d after inoculation. For the analysis, the average infection percentage of each genotype was calculated and the progenies were divided in two groups, resistant and susceptible. As the resistance phenotype was quantitative, all progenies that scored a disease level of at least 30% or higher were considered susceptible, and the genotypes with disease level less than this were considered as resistant.

Field assessment of the 106 recombinants was carried out in Oxapampa (12°34′05″ S, 75 °24′23″ W), a highland jungle agroecological zone in the Peruvian Andes with high endemic late blight pressure, which was considered a ‘hot spot’ of *Phytophthora* diversity (Gomez-Alpizar *et al.*, 2007) with multi-races (Perez *et al.*, 2001; Kaila, 2015). This is a recognized late blight resistance evaluation site of the International Potato Center’s breeding program. During the assessment, the temperature varied from 8.8 °C to 30.4 °C, with a mean of 18.4 °C, and the relative humidity varied from 38.4% to 100%, with a mean of 86.4%. The field trial was performed using the Alpha-Lattice design with three replications each consisting of 10 plants per individual. Local variety Tomasa was used as a susceptible control and 393046.7 (the original tetraploid resistance donor and parent of the population) as a resistant control. Another susceptible variety, Yungay, was planted around the field to serve as an inoculum source. After the plants started to emerge, the field was protected from late blight infection with weekly spraying of fungicide until all plants had fully emerged. After this the endemic infection was allowed to proceed, and the disease levels were evaluated weekly until the susceptible control (Tomasa) was 100% infected. The last disease evaluation (the seventh evaluation) was done on 8 December 2014. The percentage of leaf area affected was used to calculate the area under the disease progress curve (AUDPC) (Jeger and Viljanen-Rollinson, 2001) for each genotype by using the midpoint rule method (Campbell and Madden, 1990).

For sequential agroinfiltration and detached leaf late blight assays in *N. benthamiana*, the third to fifth fully expanded leaves (counted from the uppermost leaf) of 5-week-old *N. benthamiana* plants were used for agroinfiltration. Two days post*Agrobacterium tumefaciens* infiltration (dpi), plants were infected with 10 μl sporangia of *P. infestans* isolate 88069 adjusted to a concentration of 1.5 × 10^5^ sporangia ml^−1^. The droplets of sporangia suspension were inoculated onto the abaxial side of detached *N. benthamiana* leaf within the agroinfiltration site. Disease symptoms were monitored for up to 12 dpi under natural and UV light. Three replicates were conducted with at least 12 leaves in each.

### Marker development

New PCR markers were developed according to all gene sequences of the Potato Genome Sequencing Consortium (http://solanaceae.plantbiology.msu.edu/) pseudomolecule v4.03 from marker At3g24160f2 (Chr09: 58728502-58728908) to the distal end of chromosome 9. Genomic DNA samples from both parents were used as templates to amplify the polymorphism determined by PCR product length with newly developed markers of *dPI09c*. DNA of 15 resistant (AUDPC 0) and 15 susceptible (the largest 15 AUDPCs) progenies (see Supplementary Table S2), which performed no recombination in the *dPI09c* region, were selected to form resistance and susceptible pools, and were used as templates to confirm the polymorphism. The PCR products amplified by the three flanking markers (3233-1, 8384-1, and 8586-1) and four linked markers (jr38, 5455-1, jr69, and jr78-2) were sequenced and aligned to the reference genome to verify their positions and for successive bacterial artificial chromosome (BAC) screening. An overview of the newly designed primers is listed in Supplementary Table S3. Polyacrylamide (19:1) gel electrophoresis was used to separate PCR products, followed by silver staining (Li *et al.*, 2012).

### BAC library construction, screening and sequencing

A BAC library (BACGENE, Wuhan, China) was constructed with the DNA of 304413.40, one resistant clone of B3C1HP_100_. The BAC library clones were individually picked and stored in 228 384-well microtiter plates, and 384 clones in each plate were mixed to generate plate super pools. Three flanking markers, 3233-1, 8384-1, and 8586-1, and four linked markers, jr38, 5455-1, jr69, and jr78-2, were used to screen for positive super pools. Afterwards, clones in each row and column within a single plate were mixed to form row and column sub-pools. The same markers were used to locate the positive clones.

Plasmids of the positive BAC clones (clone 119 and 122) were isolated with the Qiagen Plasmid Midi Kit (Qiagen, Hamburg, Germany), and subsequently sequenced using PacBio RS II sequencing and assembled into one contig (Personalbio, Shanghai, China).

### dRenSeq analysis

Genomic DNA of resistant parent 301071.3, susceptible parent 703308, resistant bulk, and susceptible bulk consisting of 27 resistant or susceptible progenies were enriched using NB-LRR baits (Jupe *et al.*, 2013). Enrichment was followed by paired-end Illumina MiSeq sequencing and diagnostic RenSeq (dRenSeq) analysis as described previously (Van Weymers *et al.*, 2016). For characterizing the late blight resistance via dRenSeq, two genes recently identified at the end of chromosome 9, *R8* (KU530153) (Vossen *et al.*, 2016) and *R9a* (Jo *et al.*, 2015; https://www.google.com/patents/US20140041072), were added to the reference library used by Van Weymers *et al.* (2016).

### Allele mining

To confirm the presence of the complete target gene in the B3C1HP population, resistant female plant 301071.3 and two resistant progenies of B3C1HP_100_, 304413.40 and 304413.74, were subjected to PCR using *R8*-specific primers (R8-UTR_F and R8-UTR_R) (see Supplementary Table S1) followed by cloning and sequencing. Ma*R8* (the potato late blight differential of the Mastenbroek differential set Ma*R1*–Ma*R11*; Kim *et al.*, 2012; Vossen *et al.*, 2016), from which *R8* was cloned, was used as a positive control, while susceptible male plant 703308 and two susceptible progenies of B3C1HP, 304413.19 and 304413.89, were used as negative controls. Long-range PCR was conducted with Phanta Max Super-Fidelity DNA Polymerase (Vazyme Biotech, Nanjing, China) to generate blunt-end PCR products. Adenine was added to both 3′ ends, using Taq polymerase 2 µl, PCR product 36 µl, 10× buffer 5 µl, 2 mM dATP 5 µl, incubating at 72 °C for 40 min. Afterwards, PCR products were purified and cloned into pGEM^®^-T vector (Promega), and transformed to ElectroMAX™ DH10B competent cells (Invitrogen). At least eight positive clones of each genotype were sequenced and aligned with *R8* using the ClustalX 1.81 (Thompson *et al.*, 1997) and Genedoc (Nicholas *et al.*, 1997) analysis.

### Vector construction and agroinfiltration

*Avr8* (Jo, 2013) minus signal peptide was amplified from *P. infestans* isolate 88069 with attB sites to generate the entry clone, and recombined with pB7WGF2 for N-terminal enhanced green fluorescent protein (EGFP) fusion using Gateway technology. Resistance genes *R8* and *R8-like* were amplified with primer pB7-R8 (see Supplementary Table S1) and ligated into empty vector pB7WGF2, which had been digested with restriction enzyme B*sp*1407I. Recombinants were transformed into *A. tumefaciens* strain GV3101 competent cells. *R3a* and *Avr3a* (Armstrong *et al.*, 2005) plasmids harboring the late blight resistance gene *R3a* and its cognate avirulence gene, *Avr3a*, respectively, were also transformed into GV3101 as a positive control for co-infiltration tests that elicit a strong hypersensitive response, while empty vector pB7WGF2 was used as a negative control (Armstrong *et al.*, 2005).

GV3101 strains with target constructs were grown in liquid YEB medium at 28 °C overnight; bacterial cells were collected and re-suspended in modified MMA buffer (10 mM MES, 10 mM MgCl_2_, and 200 mM acetosyringone). Agrobacteria stains containing the constructs of interest were mixed and adjusted to a final OD_600_ of 0.6 and 0.3 for *R* gene and *Avr* gene, respectively. The mixed agrobacteria suspension was incubated at room temperature for 2 h in the dark before infiltration. After the incubation, a needleless syringe was used to infiltrate the agrobacteria suspension to a diffusion area of diameter 1–1.5 cm through the abaxial leaf surface. Hypersensitive responses were monitored 5 d after co-infiltration.

### Comparative genome analysis

The BAC sequences of clones 122 and 119 span together the entire QTL *dPI09c* as defined by markers 3233-1/jr38 towards the centromeric part of LG 9 and 8384-1/8586-1 towards the distal end of the chromosome. Homologous representative sequences of the *dPI09c* interval from different species including potatoes DM1-3 (DM) (Potato Genome Sequencing Consortium, 2011), 304413.40 (resistant progeny used in this study), and Ma*R8* (Vossen *et al.*, 2016), and tomatoes *Solanum lycopersicum* (Sato *et al.*, 2012) and *S. pennellii* (Bolger *et al.*, 2014) were selected for a comparative genomic study. Repeat stretches of ambiguous nucleotides (poly Ns) were removed resulting in sequences of 326207 bp in length for DM, 310320 bp for *dPI09c*, 174573 bp for *R8*, 301334 bp for *S. lycopersicum* and 557425 bp for *S. pennellii* (Supplementary Dataset S1).

Sequences were aligned using the progressive Mauve algorithm (Darling *et al.*, 2004) in the program Geneious (version 10.2) using the default condition (automatically calculate seed weight; compute locally collinear blocks (LCBs); automatically calculate the minimum LCB score; full alignment using Gapped Aligner MUSCLE3.6).

## Results

### Fine mapping of the QTL *dPI09c*

**Fig. 1.**
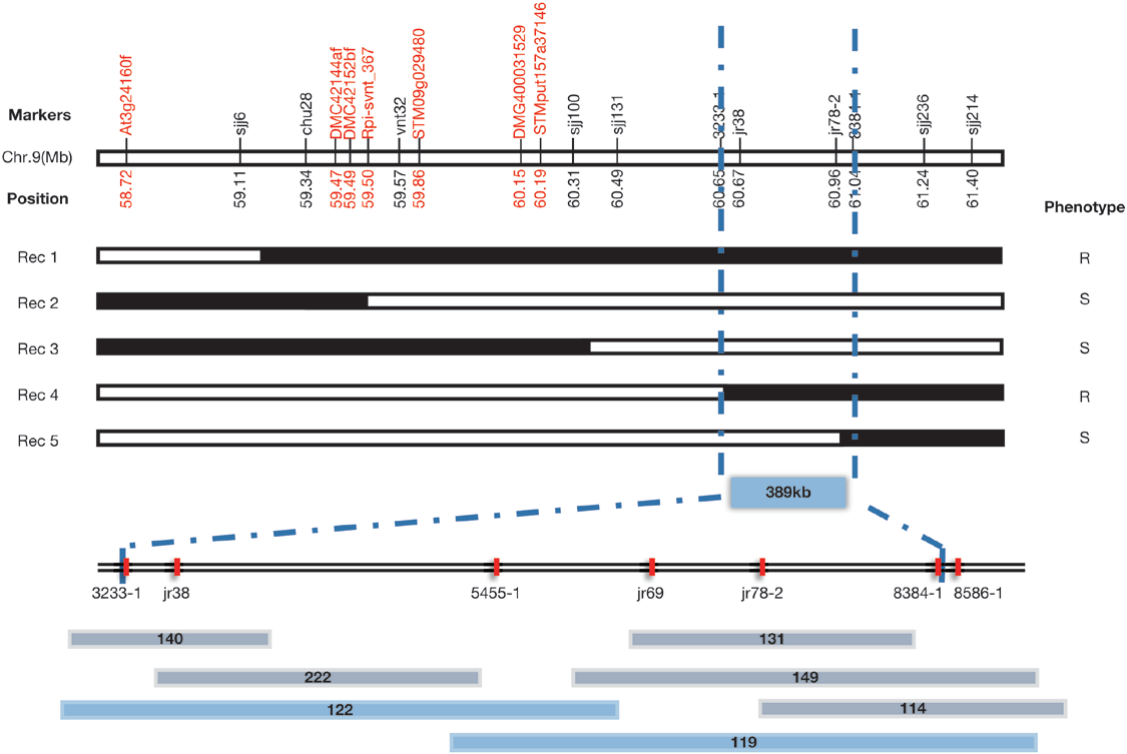
Fine mapping of *dPI09c* in population B3C1HP. Marker names and chromosomal positions are presented above and below the physical map, respectively. Marker names in red represent the markers developed in our previous study (Li *et al.*, 2012, 2015); markers in black were developed in this study. The transition between black and white rectangles indicates that the cross-over happened in the progenies. R and S represent resistance and susceptible phenotype, respectively. Rec, recombination. The vertical dashed line indicates that the resistance of *dPI09c* has been narrowed to the interval between 3233-1 and 8384-1. Red boxes show the marker position. Chromosome walking identified seven BAC clones that could cover the *dPI09c* interval. Two blue rectangles indicate BAC clones that can fully cover this interval.

Initial mapping carried out in the population B3C1HP_100_ placed the QTL *dPI09c* within the proximity of the marker DMG400031529 (Li *et al.*, 2015), which resides near *R* gene clusters with homology to *Tm-2*^*2*^ and *Sw-5* on potato linkage group 9 (Jo *et al.*, 2011, 2015; Jupe *et al.*, 2012; Li *et al.*, 2012). To fine map the resistance, a larger population comprising 4000 additional clones (B3C1HP_4000_) was assessed with initially identified markers Rpi-svnt1_367, DMC42152bf, DMC42144af, and At3g24160f2, which resulted in 106 recombinants (Fig. 1, represented by Rec 1 and 2). Further resistance assessment of these recombinants with *P. infestans* isolate PSR24 in whole-plant greenhouse tests revealed an approximate 1:1 segregation ratio of resistant recombinants that displayed less than 30% of leaf area infection with late blight and susceptible progenies that displayed more than this. Field tests revealed a similar 1:1 segregation for resistance and susceptibility (see Supplementary Table S2), which indicated a single dominant gene is concealed in this QTL. Additional markers (Supplementary Table S3) were developed towards the end of chromosome 9 based on the PGSC v4.03 pseudomolecule sequence (http://solanaceae.plantbiology.msu.edu/) at positions 58.72–61.40 Mb. We identified one recombinant between markers STMput157a37146 and 3233-1 (Fig. 1, Rec 3), and two recombinants between markers 3233-1 and 8384-1 (Fig. 1, Rec 4 and 5). Furthermore, markers jr38, 5455-1, jr69, and jr78-2 were linked to the resistance. This ultimately narrowed the locus for resistance QTL *dPI09c* to a 389 kb interval of the DM1-3 pseudomolecule sequence flanked by markers 3233-1 and 8384-1.

### Functional *R8* was identified in *dPI09c* by dRenSeq

**Fig. 2.**
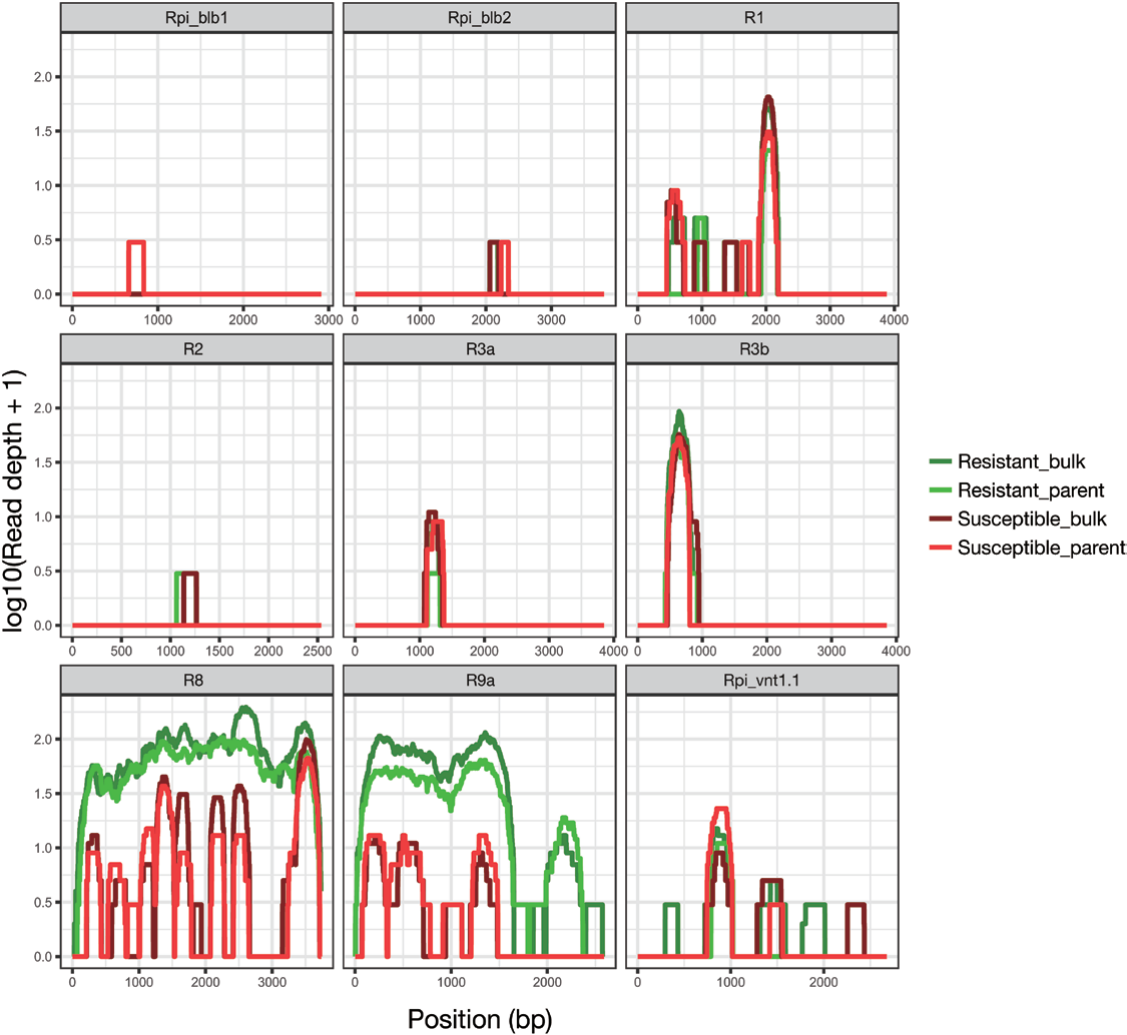
dRenSeq analysis on resistant and susceptible parent and bulks of population B3C1HP_100_. Coverage of nine functional *R* genes and read depth converted to log10 scale are depicted on the *x*-axis and *y*-axis. Light green and dark green curves represent resistant parent and bulk, red and scarlet curves represent susceptible ones, respectively. A high stringent mismatch rate (0.5%) was used for read mapping.

The fine mapping of the resistance in the B3C1HP_4000_ population placed the QTL *dPI09c* in a genomic region that is known to contain a number of functional NB-LRRs such as *R8*, *R9a*, *Rpi_moc1*, and *Rpi_vnt1* (Smilde *et al.*, 2005; Foster *et al.*, 2009; Pel *et al.*, 2009; Jo *et al.*, 2015; Vossen *et al.*, 2016). To ascertain if a known NB-LRR gene could explain the resistance in the *dPI09c* QTL, we conducted a dRenSeq analysis (Van Weymers *et al.*, 2016). Genomic DNA of resistant parent 301071.3, susceptible parent 703308, and bulks consisting of 27 resistant and 27 susceptible progenies of the B3C1HP_100_ population were enriched using NB-LRR baits (Jupe *et al.*, 2013). Reads were mapped, using a high stringent 0.5% mismatch rate, against nine functional late blight NB-LRR genes, namely *Rpi-blb1*, *Rpi-blb2*, *R1*, *R2*, *R3a*, *R3b*, *R8*, *R9a*, and *Rpi-vnt1.1*. Under these conditions, the RenSeq reads only map to the reference set containing functional NB-LRRs if the reads have a maximum of one SNP in 200 bp of sequence compared with the reference. Mapping results demonstrated that the reads of the resistant parent and resistant bulk generated full coverage of *R8*, while only partial coverage was achieved using reads from the susceptible parent or the susceptible bulk (Fig. 2). The read depth is an important indication of the completeness of the coverage, as a partial coverage in susceptible parent and bulk indicates that some parts of the functional gene *R8* are conserved. These results strongly suggest that *R8* could be a main contributor towards the function of *dPI09c* for late blight resistance.

### The presence of *R8* was confirmed by allele mining

To confirm the presence of a complete and intact *R8* gene in the B3C1HP_100_ population, *R8*-specific primers (Supplementary Table S1) were utilized to amplify a 7 kb fragment that encompasses functional *R8* (including coding and regulatory sequences). We included the susceptible male parent and two susceptible progenies (304413.19 and 304413.89) as negative controls, and Ma*R8* as a positive control. As expected, all susceptible plants did not yield the *R8*-specific amplicon (see Supplementary Fig. S1). The PCR products of resistant female parent and two resistant progenies were cloned and sequenced. Our analysis confirmed that all clones contained the *R8* gene and no sequence variation was identified (data not shown).

### *R8* and functional *R8-like* resistance is found in diverse breeding material and wild species

**Fig. 3.**
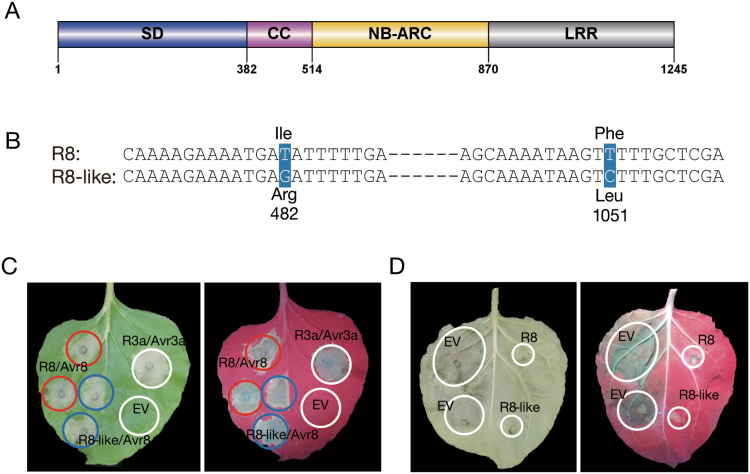
The point mutations of *R8-like* in *S. demissum* does not change its function. (A) Schematic representation of R8 protein. SD, Solanaceae domain; CC, coiled-coiled domain; NB-ARC, nucleotide-binding adaptor shared by APAF-1, R proteins, and CED-4 domain; LRR, leucine rich repeat domain. (B) The alignment of *R8* and *R8-like* on the two point mutation fragments. The mutated bases are shaded. (C) Transient overexpressing R8-like/Avr8 shows a similar level of hypersensitive response as R8/Avr8. R3a/Avr3a was used as positive control, and empty vector (EV) pB7WGF2 as negative control. The image is representative of three biological repeats taken at 5 dpi under natural and UV light. (D) Leaf images under natural and UV light showing the *P. infestans* colonization on *N. benthamiana* 12 dpi. (This figure is available in color at *JXB* online.)

To ascertain if *R8* is also present in additional late blight resistance resources, we used PCR to test previously identified sources of late blight resistance that were obtained following late blight assays with highly aggressive *P. infestans* isolates UK3928A in the UK (Van Weymers *et al.*, 2016) and HB14-2 and HB16-2 collected in infected fields in Hubei province of China. Thirty-two out of 242 tested genotypes showed resistance to the pathogens, with three susceptible cultivars (Yungay, E-Potato 3, and Huashu 1) being utilized as negative controls (see Supplementary Table S4). The results showed that 21 resistant plants putatively contained *R8* as they amplified the expected 7 kb fragment. These included 15 progenies descending from the B3 population, four cultivars (06HE13-1, 08HE171-1, 08HE171-6, and E-Potato 5), and two wild species (*Solanum phureja* accession IVP196-2 and *S. demissum* accession CT9-4). As expected, none of the susceptible plants yielded the *R8*-specific amplicon. PCR products of seven out of the 15 progenies from the B3 population and all additional putative *R8*-containing genotypes were cloned and at least eight recombinant clones from each plant were sequenced. There was no sequence variation in *R8* alleles except for the wild species, *S. phureja* accession IVP196-2 and *S. demissum* accession CT9-4. In *S. phureja* IVP196-2, several insertions and deletions in the promoter were evident alongside many SNPs and a premature stop codon that results in a pseudogenized gene (see Supplementary Fig. S2). *Solanum demissum* accession CT9-4 has a complete coding sequence like *R8* (Fig. 3A) except for two non-synonymous SNPs at position 482 and 1051 in the Solanaceae domain (SD) that changed the amino acids from Ile to Arg and from Phe to Leu, respectively (Fig. 3B) and is subsequently referred to as *R8-like*.

To investigate whether *R8-like* is a functional *R* gene, it was transiently co-expressed with the cognate avirulence gene of *P. infestans*, *Avr8*, in the model Solanaceae plant *N. benthamiana*, as shown by Vossen *et al.* (2016). Co-infiltration-specific cell death, which is indicative of a recognition response, was evident upon co-infiltration of a resistance gene and an avirulence gene such as the positive control *R3a*/*Avr3a*, while the empty vector control pB7WGF2 showed no such phenotype. Importantly, this response between *R8-like* and *Avr8* was phenotypically not distinct from the cell death that was elicited after the co-infiltration of *R8* with *Avr8* 5 d post-infiltration (Fig. 3C). Five-week-old *N. benthamiana* leaves transiently expressing *R8* and *R8-like* were challenged with *P. infestans* isolate 88069. *R8-like* was demonstrated to be a functional *R* gene as it efficiently stopped colonization by *P. infestans* (Fig. 3D).

### Genomic analysis of *dPI09c*

In order to investigate the genomic organization of the *dPI09c* locus, a resistant progeny of B3C1HP_100_, 304413.40, was used to construct a BAC library. The library consisted of 87552 recombinant clones with an average insert size of 110 kb, thus covering the genome 10-fold. The BAC library was screened with seven PCR markers of the 389 kb region in DM, and in total seven positive BAC clones were identified (Fig. 1). The BAC clones 122 and 119 fully covered the *dPI09c* interval and were subsequently sequenced. The assembled contig is 310.32 kb in length. Importantly, the contig contains the entire *dPI09c* interval flanked by markers 3233-1 and 8384-1, which is 186 kb in length and therefore shorter than the 389 kb region predicted in the potato reference genome.

**Fig. 4.**
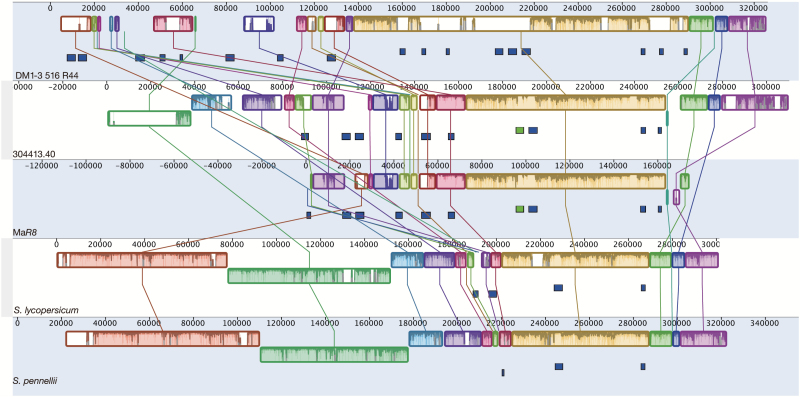
Genomic comparison of *dPI09c* interval among potato DM1-3 516 R44 (Potato Genome Sequencing Consortium, 2011), 304413.40 (Resistant progeny used in this study) and Ma*R8* (potato late blight differential of Mastenbroek differential set Ma*R1*–Ma*R11*; Vossen *et al.*, 2016), and tomato *Solanum lycopersicum* (Sato *et al.*, 2012) and *S. pennellii* (Bolger *et al.*, 2014). The large blocks in different colors show the homology of the genome. The small green rectangles beneath the large blocks represent *R8*, blue rectangles are *R8* analog, and forward and reverse direction of the analogs are indicated by the upper and lower rectangles, respectively. Sequences were aligned using progressive Mauve algorithm (Darling *et al.*, 2004) in the program Geneious (version 10.2) with default settings.

Synteny analysis among homologous representative sequences of the *dPI09c* interval from potatoes DM (Potato Genome Sequencing Consortium, 2011), 304413.40 (resistant progeny used in this study) and Ma*R8* (Vossen *et al.*, 2016), and tomatoes *S. lycopersicum* (Sato *et al.*, 2012) and *S. pennellii* (Bolger *et al.*, 2014) demonstrated rearrangements between all the haplotypes from different species in this interval, while a high homology between both Ma*R8* and 304413.40 was depicted (Fig. 4). Genomic sequence comparison between Ma*R8* and 304413.40 revealed that the same fragment has been introduced into different cultivars, suggesting that the resistance that *dPI09c* is conferring might be derived from a common *S. demissum* source. Nevertheless, there is a slight difference between the two *R8*-containing haplotypes at both BAC ends. The two analogs of *R8* in the front end of Ma*R8* BAC and 304413.40 BAC shared less than 40% sequence identity, and the similarity between the flanking sequence was very low as well (data not shown). This suggests either this is a breaking point of recombination in different haplotypes or there was incomplete sequencing of the haplotype. *R8* paralog copy numbers vary in haplotypes from different species, with 17 in DM, 10 in 304413.40 and Ma*R8*, four in *S. lycopericum*, and three in *S. pennellii*. However, the *R8* sequence is somewhat conserved comparing between the species. These results suggested that *R8* progenitors are most likely ancient genes that have undergone distinct evolution in different species.

## Discussion

In a previous study, we have identified the late blight quantitative resistant QTL *dPI09c* (Li *et al.*, 2012) as a major QTL located at the end of the chromosome 9. Here, we have taken a map-based cloning approach to narrow the genetically defined interval to a 389 kb fragment with new markers 3233-1 and 8384-1 designed based on the reference genome of potato (Fig. 1). In this region an array of late blight *R* genes including *Rpi-moc1* (Smilde *et al.*, 2005), *Rpi-vnt1* (Foster *et al.*, 2009), *R8* (Jo *et al.*, 2011), *R9a* (Jo *et al.*, 2015), and *Ph-3* (Zhang *et al.*, 2014) have been reported. Using the diagnostic RenSeq method (dRenSeq), we identified the full coding sequence of *R8* in the resistant progenitor and the bulk consisting of resistant progenies. (Fig. 2). Allele mining confirmed that the resistance gene concealed in *dPI09c* is identical to *R8* of *S. demissum* (Vossen *et al.*, 2016). This was further confirmed by BAC library screening and sequencing, which also enabled a comparative genome study and indicated that this interval is conserved in other Solanaceae haplotypes.

This study provides clear evidence that a single major disease resistance gene can explain the QTLs, as has previously been speculated. Indeed, cultivars such as Sarpo Mira display field resistance-type responses to late blight and are now known to also contain *R8* but in combination with other major *R* genes (Rietman *et al.*, 2012; Vossen *et al.*, 2016). Plants of the B3C1HP population containing *R8* generally show expanding lesions in the detached leaf assay (data not shown), and in the B3 population the resistance in severe epidemics is consistent with what had been considered a quantitative type (Landeo *et al.*, 1999; Lindqvist-Kreuze *et al.*, 2014). CIP’s B3 population incorporated a battery of resistant resources, including *S. demissum*-derived advanced sources of population A, native cultivars from *S. phureja*, *Solanum andigena* adapted to long days (Neotuberosum), and combined material of *Solanum acaule*, *Solanum bulbocastanum*, and *S. tuberosum* (ABPTs) through four-way hybrids (Landeo, 1989). An attempt was made to rule out major *R* genes in this population by selecting only progenies that were not fully resistant three decades ago (Landeo *et al.*, 1995). The quantitative resistance phenotype of B3C1HP is probably the reason why the responsive gene of *dPI09c*, *R8*, was retained in the B3 population after stringent screenings. Thus, this study significantly shows how difficult it can be to select progenies against the presence of major *R* genes. Whether minor contributors to *R8* reside in the genetically defined interval may be studied in the future as *R8*-based resistance is highly dependent on the genetic background as described by Vossen *et al.* (2016) and in our detached leaf assay (data not shown). Hence, mining the *R8* gene stability and functionality regulator or other defense-related genes could also assist with the resistance breeding program in the future.

Some QDRs have been identified as co-localizing with a major *R* gene locus. Three QTLs conferring resistance to the powdery mildew *Oidium lycopersici* were found adjacent to qualitative loci, with *Ol-qtl1* on chromosome 6 in the same region as the *Ol-1*, *Ol-qtl2*, and *Ol-qtl3* on chromosome 12 in the vicinity of the *Lv* locus conferring resistance to another powdery mildew species, *Leveillula Taurica* (Bai *et al.*, 2003). It has been reported that potato late blight QDR on chromosome 5 co-localizes with *R1* (Collins *et al.*, 1999; Beketova *et al.*, 2006). However, it is likely that the quantitative resistance on chromosome 5 is not caused by QDR but by maturity type, which is linked to *R1* (Collins *et al.*, 1999; Beketova *et al.*, 2006). In addition, the effects of ‘defeated’ or ‘weak’ *R* genes have been reported in many plants with quantitative disease resistance (Poland *et al.*, 2009; Kou and Wang, 2010; St Clair, 2010; Roux *et al.*, 2014; French *et al.*, 2016). For example, the rice bacterial blight disease resistance gene *Xa4* has been regarded as a ‘defeated *R* gene’ that confers resistance to multiple strains of *Xanthomonas oryzae pv. oryzae* (*Xoo*) (Li *et al.*, 1999). In potato, QDR to late blight has been found durable in the B3 population, Sarpo Mira, Stirling, and other cultivars (Solomon-Blackburn *et al.*, 2007; Rietman *et al.*, 2012; Lindqvist-Kreuze *et al.*, 2014). In the present study, the cloning of *R8* provides strong evidence that quantitative resistance can be caused by an NB-LRR gene, which is normally thought to be responsible for qualitative resistance. Besides, *S. demissum* differential Ma*R8*, Ma*R9*, and Ma*R10* have also shown broad-spectrum and quantitative resistance in the field (Bradshaw *et al.*, 2006; Jo *et al.*, 2011; Xu *et al.*, 2013; Jo *et al.*, 2015), which suggests that when novel field resistance is identified, we must be careful with the assumptions made about the molecular basis of such resistance and cannot rule out major *R* genes as the main contributors. Helpfully, with the recently developed dRenSeq method, field resistance of potato materials can be quickly detected to check if known *R* genes exist, which is an efficient way to avoid time-consuming map-based cloning. Also, dRenSeq has a potential to investigate not only late blight resistance genes but also other significant traits, such as potato resistance to cyst nematodes, viruses, and bacteria.

NB-LRR genes are well known to be clustered in plant genomes by tandem and segmental duplications (McDowell and Simon, 2006). Functional homologs have been confirmed in late blight *R* gene clusters such as *R2*, *R3a*, *R3b*, and *Rpi-vnt1* loci (Huang *et al.*, 2005; Foster *et al.*, 2009; Lokossou *et al.*, 2009; Pel *et al.*, 2009; Li *et al.*, 2011; Lenman *et al.*, 2016). In this study, we dissected that *R8* is located in such an *R* gene cluster. We identified a functional *R8* homolog as defined by *R8-like* in *S. demissum* with two non-synonymous amino acid mutations (Fig. 3). These could result from random point mutations in different *S. demissum* accessions, or be a consequence of pathogen selection pressure in natural environments that might lead to novel effector recognition, but this is not clear at present. Nonetheless, sequence alignment revealed that a similar fragment has been introduced to Ma*R8* and 304413.40 from *S. demissum* (Fig. 4), suggesting that scientists might exploit the same *S. demissum* resources (Black *et al.*, 1953; Malcolmson and Black, 1966) for resistance breeding against late blight. However, the BACs of 304413.40 for sequencing only contain the resistant haplotype derived from the resistant female parent 301071.3; little is known about the sequence of the susceptible one. There were no amplicons obtained when amplifying *R8* in susceptible plants. Therefore, the difference between the resistant and susceptible haplotypes is speculated to be the sequence variations in the 5′-untranslated region (UTR) or the 3′-UTR of *R8*, as the *R8*-specific primers were designed to amplify the full length of *R8* only. How *R8* might have evolved in the *dPI09c* locus between the two haplotypes remains to be established, if the susceptible BAC clones can be identified and sequenced. Genomic comparison among Solanaceae species showed significant rearrangements in the *dPI09c* interval. However, R8 shares 83.3% identity with its ortholog Sw-5b in tomato (Vossen *et al.*, 2016) and is relatively conserved compared with other analogs in Solanaceae. These findings suggest that *R8* analogs probably originated from the same ancestor and underwent distinct evolution in response to diverse challenges.

The *R8* gene was identified in some resistant potato cultivars (see Supplementary Table S4) indicating its feasibility in the resistance improvement to cope with unexpected environmental and pathogen changes. This *R* gene with quantitative features of resistance has been observed to combat a broad range of pathogens (Roux *et al.*, 2014) and is considered to be a generalist. *Sw-5b*, the ortholog of *R8* in tomato (Brommonschenkel *et al.*, 2000; Spassova *et al.*, 2001), which has the SD (Mucyn *et al.*, 2006; Chen *et al.*, 2016), is a versatile *R* gene and confers broad resistance against tospoviruses, including *Tomato spotted wilt virus*, *Groundnut ring spot virus*, and *Tomato chlorotic spot virus* (Boiteux and Giordano, 1993; Bendahmane *et al.*, 2002). Mi-1, also containing the SD domain, which is thought to have dual regulatory roles of activating the Mi-1 resistance protein (Lukasik-Shreepaathy *et al.*, 2012), is responsible for the resistance against root-knot nematodes, whitefly, and aphids (Rossi *et al.*, 1998; Vos *et al.*, 1998; Nombela *et al.*, 2003). Whether *R8* can bring resistance to other pathogens like these SD-encoding *R* genes and whether the mechanism underlying the quantitative resistance is due to the dynamic allele variation (Fig. 3) should be intriguing areas for further study.

It has been argued that *R* gene stacking is by far the best strategy to improve late blight quantitative resistance in the field (Stewart *et al.*, 2003; Zhu *et al.*, 2012). We know that some of the major *R* genes are more likely to be defeated as a result of strong selection pressure for the cognate fast evolving effectors, for instance, the evading form of Avr3a^EM^ (Armstrong *et al.*, 2005), the truncated Avr4 (van Poppel *et al.*, 2008), and the expression-reduced Avrvnt1 (Pel, 2010; Vleeshouwers *et al.*, 2011). However, *R8*, *R9a*, *R10*, and *Rpi-blb1* still maintain their quantitative resistance. *R* genes that confer quantitative disease resistance to late blight are more likely to be durable and are presumed to have less selection pressure on pathogens; they may be good candidates for *R* gene stacking. This novel way of stacking resistance genes may be a great achievement for the breeding community in maintaining the durability of potato resistance against late blight and increase the life expectancy of potato cultivars. Also, it may be technically possible for several of these gene stackings to induce high level resistance. Molecular markers are needed for every contributing QTL, including functional markers, such as R/Avr gene responses, to help in rapid and precise selection of resistance in breeding lines.

## Supplementary data

Supplementary data are available at *JXB* online.

Fig. S1. *R8* gene allele mining in population B3C1HP_100_.

Fig. S2. Schematic alignment of *R8* in 301071.3 and IVP196-2.

Table S1. The list of primers used in this study.

Table S2. Late blight phenotype of B3C1HP_106/4000_ tested in greenhouse by *P. infestans* inoculation and in the field by natural disease infection in 2014.

Table S3. Polymerase chain reaction markers newly developed in this study.

Table S4. The list of 32 resistant materials for allele mining.

Dataset S1. The FASTA-file for genomic sequences of *dPI09c* interval in potato DM1-3 516 R44 (Potato Genome Sequencing Consortium, 2011), 304413.40 (resistant progeny used in this study), Ma*R8* (potato late blight differential of Mastenbroek differential set Ma*R1*–Ma*R11*; Vossen *et al.*, 2016), and tomato *S. lycopersicum* (Sato *et al.*, 2012) and *S. pennellii* (Bolger *et al.*, 2014).

Supplementary Tables and Figures

Supplementary Table2

Supplementary Data

## Abbreviations:

dpi: days post inoculation
dRenSeq: diagnostic RenSeq (resistance gene enrichment sequencing)
QDR: quantitative disease resistance;
SD: Solanaceae domain.
