# Supplementary material for: Potato late blight field resistance from QTL dPI09c is conferred by the NB-LRR gene R8
Source: J Exp Bot. 2018 Jan 27;69(7):1545–55. doi: 10.1093/jxb/ery021 (PMC5889011; doi:10.1093/jxb/ery021)
Supplement: Supplementary Tables and Figures [file ery021_suppl_supplementary_tables_and_figures.pdf]

Supplementary Figure S1.

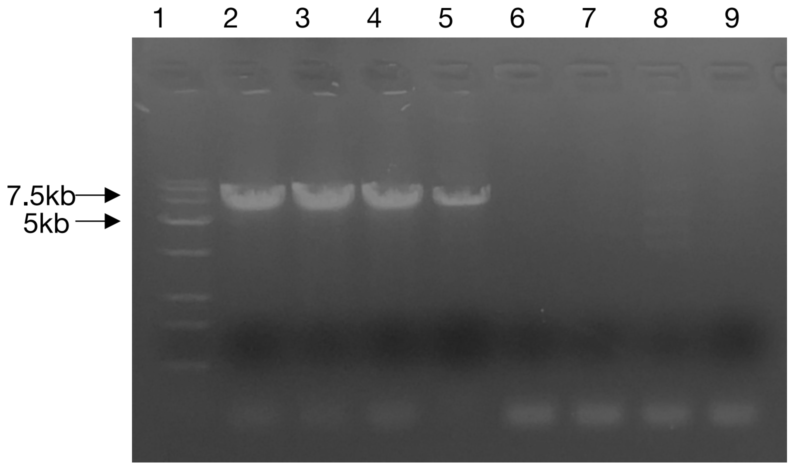

Fig. S1. *R8* gene allele mining in population $B3C1HP_{100}$. PCR amplicons (7kb) of individuals in population $B3C1HP_{100}$ were obtained using *R8*-specific primers, marker size is indicated by arrow. Lane 1-9: Marker 15K (Transgen biotech), *R8* amplicons from 301071.3 (resistant female parent), 304413.40, 304413.74, Ma*R8* (positive control), 703308 (susceptible male parent), 304413.19 and 304413.89, NTC (no template control).

Supplementary Figure S2.

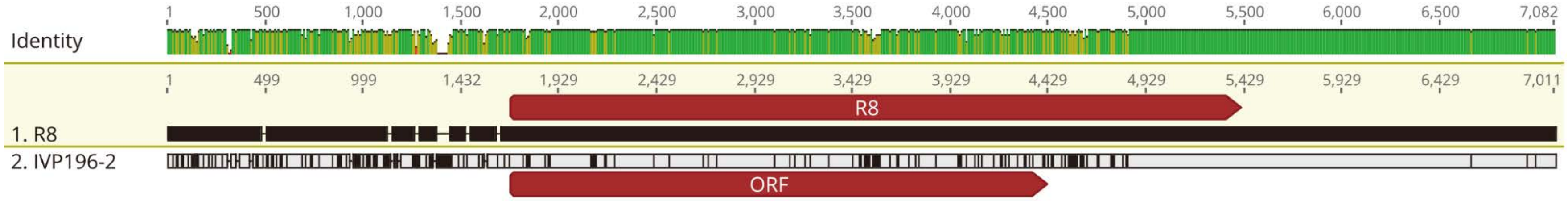

Fig. S2. Schematic alignment of *R8* in 301071.3 and IVP196-2. The full length DNA sequence of *R8* is in black; the amplicon in IVP196-2 is in gray, the black vertical lines represents the difference from *R8*. Putative open reading frames from the two materials are indicated by red arrows.

Supplementary Table S1. The list of primers used in this study

Marker name	Primer	Usage	Reference
At3g24160f2_F	AGAGCAATATGGAGCAGAAGGTGTTG	Fine mapping	Li et al., 2012
At3g24160f2_R	TCGAATAATGGCTCAGCTACTGAAGG		
Rpi_svnt1_367_F	ATCTATCCACAGCACGTGTTG	Fine mapping	Li et al., 2012
Rpi_svnt1_367_R	ACATCACCTGCCAGTTGTTG		
DMC42152bf_F	CCGGCCCCTTAACTTGGCCCA	Fine mapping	Li et al., 2012
DMC42152bf_R	AGCACCCTCCAAGCTCACGAAAGTT		
DMC42144af_F	ACACCGCCGAGAAAGTCCAGCA	Fine mapping	Li et al., 2012
DMC42144af_R	ATTTGATCTAGAACGTGCCCTGAGC		
DMG400031529_F	ATAAACAATCTATTTGTCACCCCTAC	Fine mapping	Li et al., 2015
DMG400031529_R	CCTCCATACCAACCACCTCTA		
STM09g029480_F	AACACCCTTCTGTTGGTTGC	Fine mapping	Li et al., 2015
STM09g029480_R	CGCCATTCTTCCAAACATCT		
STMput157a37146_F	GTGTTGGTGGCGGATACC	Fine mapping	Li et al., 2015
STMput157a37146_R	CCACGGTTGCCTGAGTATTT		
R8-UTR_F	AAAACTTTCACGCACCCATAGGA	*R8* gene allele mining	Vossen et al., 2016
R8-UTR_R	AACAAGAGATGAATTAAGTCGGTAGC		
pB7-R8_F	TCGGCATGGACGAGCTGTACAAGATGAATGAAAATGAAATTGAGGAA	Recombinant cloning of *R8*	This study
pB7-R8_R	GGGATATCACCACTTTGTACATCAATCTCTTCGACTTCTTCTTACG		
attB1-Avr8_F	AAAAAGCAGGCTTCACCATGACACCAGCACCGCCACAAGTAT	Gateway cloning of *Avr8*	This study
attB1-Avr8_R	AGAAAGCTGGGTCTTACGATGTTTTCGCTTCTTTAA		
attB1	GGGGACAAGT TTGTACAAAAAAGCAGGCT	Adaptor for gateway cloning	Invitrogen
attB2	GGGGACCACTTTGTACAAGAA AGCTGGGT		

Supplementary Table S3. Polymerase Chain Reaction (PCR) markers newly developed in this study.

Marker name	Physical position in DM pseudomolecule	Primer	Tm (°C)
sjj6F	Chr09:59118740-59123025	GCTTCAATCTTATTTCTACCA	56
sjj6R		TTTTCACCACACCAACTTCTG	
vnt32F	Chr09:59573146-59575193	CACATGCTGTGGATGAGATAC	56
vnt32R		ATAAGAACTGCCTTTCGACTT	
sjj100F	Chr09:60308417-60310209	TCTCCCTCATTGCTATTTTG	56
sjj100R		TGTTGTTCTCGTTGTTGGTC	
sjj131F	Chr09:60496212-60498073	GGGTTTTTGGTAAGTGTGTG	57
sjj131R		CTCGACTGCTGTTATTGGAT	
3233-1F	Chr09:60652736-60653825	GAGCGATAGAGCCCTTCCACAC	55
3233-1R		AGGAGATCCCCCTTAGCTTTGC	
jr38F	Chr09:60677124-60678464	AATCCAGAAAGCTCCATCCAC	55
jr38R		CTTTTGCCTGATTCCAGAGAC	
5455-1F	Chr09:60829857-60830729	ATTGTTCCTTCGCTTTGCTTG	55
5455-1R		ACCACTCTTCTAAACGTGGGT	
jr69F	Chr09:60902146-60905918	GTGCGAAGAATATAGTCTGGC	55
jr69R		TCAGCAAAGCTAAGTGGGATC	
jr78-2F	Chr09:60956907-60960662	CGGGAGGAACGAAGGAACTAG	55
jr78-2R		AAACACGGGACTTTGGACGAG	
8384-1F	Chr09:61041590-61042811	ATAGGAGGTTGAGCAACGTGAA	55
8384-1R		GGCTGTAGGTGAGAGAGGGTAT	
8586-1F	Chr09:61050190-61051170	AGTTTACCCATTTCCCATTCC	55
8586-1R		CCCCGGTTGTAGTTTATGTTC	
sjj236F	Chr09:61240781-61241429	AGCCAAAGCAAGTTCAAAT	56
sjj236R		ACAGAAGCCACAACACAAA	
sjj214F	Chr09:61398739-61402533	GGGTTTGCCATTGTTTAG	56
sjj214R		AGAGCGGAGGAGAGTTTT	

Supplementary Table S4. The list of 32 resistant materials for allele mining.

No.	Name	Origin	*R8*	No.	Name	Origin	*R8*
1	391585.179	CIP B3C1[a]	Yes	19	7650	CPC, *S. bulbocastanum*	No
2	392617.54	CIP B3C1	Yes	20	7620	CPC, S. canasense	No
3	392634.52	CIP B3C1	Yes	21	7129-1	CPC, *S. okadae*	No
4	395017.242	CIP B3C2[b]	Yes	22	7129-2	CPC, *S. okadae*	No
5	395096.2	CIP B3C2	Yes	23	CT9-4	*S.demissum*	Yes
6	395112.9	CIP B3C2	Yes	24	SD-14	*S.demissum*	No
7	395114.5	CIP B3C2	Yes	25	SD-27	*S.demissum*	No
8	395123.6	CIP B3C2	Yes	26	SD-46	*S.demissum*	No
9	396018.241	CIP B3C2	No	27	IVP196-2	*S.phureja*	No
10	BP05-8	HZAU B3C2[c]	Yes	28	J101K27	Somatic hybrid,<br>*S. tuberosum+S. bulbocastanum*	No
11	BP10-2	HZAU B3C2	Yes	29	06HE13-1	Chinese cultivar	Yes
12	BP14-1	HZAU B3C2	Yes	30	08HE171-1	Chinese cultivar	Yes
13	BP14-101	HZAU B3C2	Yes	31	08HE171-6	Chinese cultivar	Yes
14	BP16-73	HZAU B3C2	Yes	32	E-Potato 5	Chinese cultivar	Yes
15	BP18-36	HZAU B3C2	Yes	NC[e]	Yungay	Peruvian cultivar	No
16	BP22-32	HZAU B3C2	Yes	NC	E-Potato 3	Chinese cultivar	No
17	7636	CPC[d], *S. bulbocastanum*	No	NC	Huashu 1	Chinese cultivar	No
18	7638	CPC, *S. bulbocastanum*	No				

[a] CIP' B3 population was improved for one recurrent selection (B3C1, B3 cycle 1) (Landeo *et al*., 1999).

[b] CIP' B3 population was improved for two recurrent selections (B3C2, B3 cycle 2) (Landeo *et al*., 1999).

[c] Seventeen B3C1 clones were successfully crossed with bulk pollen which was mixed with approximately equal amount of pollens from 27 B3C1 clones. Seeds produced were used in further selections to form the offspring population HZAU B3C2 (Yao *et al*., 2011).

[d] CPC, Commonwealth Potato Collection.

[e] NC, negative control

Reference

Landeo J, Gastelo M, Beltran G, Diaz L. 1999. Quantifying genetic variance for horizontal resistance to late blight in potato breeding population B3C1. CIP program report **2000**, 63-68.

Yao CG, Song BT, Liu J, Wu CJ, Cheng Q, Li DC, Xie CH. 2011. Population improvement of resistance to late blight in tetraploid Potato: A case study in combination with AFLP marker assisted background selection. Agricultural Sciences in China **10,** 1177–1187.
